# Embedding international medical student electives within a 30-year partnership: the Ghana-Michigan collaboration

**DOI:** 10.1186/s12909-020-02093-6

**Published:** 2020-06-12

**Authors:** Emma R. Lawrence, Cheryl Moyer, Carrie Ashton, Bolade A. R. Ibine, Nauzley C. Abedini, Yaera Spraggins, Joseph C. Kolars, Timothy R. B. Johnson

**Affiliations:** 1 Department of Obstetrics and Gynecology, University of Michigan, 1500 E. Medical Center Dr, Ann Arbor, MI 48109 USA; 2 Learning Health Sciences, University of Michigan, 1111 E. Catherine St, Ann Arbor, MI 48109 USA; 3 Global REACH, University of Michigan, 1111 E. Catherine St, Ann Arbor, MI 48109 USA; 4 Department of Obstetrics and Gynecology, Family Health University College, PO Box TS 669 Teshie, Accra, Ghana; 5 Division of Palliative Medicine, University of California, San Francisco, 533 Parnassus Ave, San Francisco, CA 94143 USA; 6 College of Literature, Science and Arts, University of Michigan, 500 S. State St, Ann Arbor, MI 48109 USA; 7 Department of Internal Medicine, University of Michigan, 1500 E. Medical Center Dr, Ann Arbor, MI 48109 USA

**Keywords:** International medical student electives, Global education partnership, Bidirectional exchange

## Abstract

**Background:**

Global health experiences are an increasingly popular component of medical student curricula. There is little research on the impact of international medical electives embedded within long-standing, sustainable partnerships. Our research explores the University of Michigan medical student elective experience in Ghana within the context of the Ghana-Michigan collaborative.

**Methods:**

Study participants are University of Michigan medical students who completed an international elective in Ghana between March 2006 and June 2017. Post-elective reports were completed by students, including a description of the experience, highlights, disappointments, and the impact of the experience on interest in future international work and future practice of medicine. A retrospective thematic analysis of reports was carried out using NVivo 12 (QSR International, Melbourne, Australia).

**Results:**

A total of 57 reports were analyzed. Benefits of the elective experience included building cross-cultural relationships, exposure to different healthcare environments, hands-on clinical and surgical experience, and exposure to different patient populations. Ninety-five percent of students planned to engage in additional international work in the future. Students felt that the long-standing bidirectional exchange allowed them to build cross-cultural relationships and be incorporated as a trusted part of the local clinical team. The partnership modeled collaboration, and many students found inspiration for the direction of their own careers.

**Conclusions:**

Embedding clinical rotations within a well-established, sustained partnerships provides valuable experiences for trainees by modeling reciprocity, program management by local physicians, and cultural humility—all of which can help prepare learners to ethically engage in balanced, long-term partnerships in the future.

## Background

Global health experiences are an increasingly popular component of medical student curricula. According to the 2019 Association of American Medical Colleges (AAMC) Medical School Graduation Questionnaire, 29% of University of Michigan medical students and 24% of all graduating medical students participated in a global health experience. Interviews with medical students demonstrate that the majority of students believe that global electives are beneficial to medical education [1].

Past research suggests that participation in international medical electives is associated with improvement in clinical skills, increased knowledge of tropical medicine, and careers in underserved or primary care settings [1]. Students also gain cultural competency and increased awareness of social determinants of health [2–4]. However, criticisms of international electives—both at the individual and institutional level—is the burden that visiting American students place on low-resource health systems, the lack of bidirectional training opportunities for trainees from host countries, and a lack of longitudinal pre- and post-departure engagement [1, 5, 6]. Recommendations have been made for ethical engagement in international electives, including sustainable approaches to preparation and debriefing, systems of reciprocity, and overarching structures of long-term institutional engagement. However, adoption remains low [7].

Few studies have explored the impact of participation in international medical electives embedded within long-standing, sustainable partnerships. Beginning with the co-creation of an in-country post-graduate training program for Ghanaian obstetrician gynecologists (OBGYNs) in 1989, the University of Michigan Medical School (UMMS) has had long-standing relationships with a growing number of partner institutions in Ghana [8–10], where it has been intentional about developing an ethically informed bidirectional partnership between the University and collaborative Ghanaian medical schools [11, 12]. In addition to the 246 OBGYN residents trained to date, a hallmark of the partnership has been a robust bilateral exchange of learners and faculty from multiple medical specialties. Our previous research outlined the impact of this collaboration on Ghanaian trainees [8, 9, 13]. What has not been explored is the unique impact on UMMS students within the parameters of this sustained 30-plus year partnership. Our current research examines the University of Michigan medical student elective experience in Ghana within the context of the Ghana-Michigan collaborative.

### Ghana-Michigan partnership background

The University of Michigan has a long-established relationship with Ghana for medical education—the foundation of which is built on a Ghana-based post-graduate OBGYN training program. There have been 246 OBGYN physicians trained in-country since 1989, with retention of 236 certified specialists [14–17]. Bilateral senior resident exchanges have been established since 1989 between UMMS and two teaching institutions [1] in Ghana to strengthen the development of OBGYN residency training programs there [14, 15]. Michigan-Ghana partnerships in education have expanded to develop Ghana-based post-graduate training programs in Emergency Medicine, Family Medicine, Psychiatry, Pediatric Ophthalmology, and Audiology.

Since 2007, UMMS has facilitated a bilateral medical student exchange program between senior medical students at UMMS and universities in Ghana. This exchange was developed to increase opportunities for UMMS students to participate in international learning experiences, and to respond collaboratively to Ghanaian partners who wanted new experiences at American institutions [9].

All interested UMMS students are accepted for international electives. UMMS students typically spend 4 weeks at an academic hospital in Ghana as an elective rotation in their final year of medical school. Long-standing relationships have been established at the Korle Bu Teaching Hospital in Accra and the Komfo Anokye Teaching Hospital in Kumasi. The majority of students rotate in OBGYN due to its long-established status, but depending on individual interests, may also rotate in other specialties including General Surgery, Internal Medicine, and Pediatrics.

A memorandum of understanding was created between UMMS and partner schools in Ghana, asserting that neither Ghanaian students nor UMMS students are charged tuition for participation in their elective. Costs for the international elective for UMMS students include airfare, visa, health insurance, vaccinations, local housing, local transportation, and food. Local housing is provided at the medical student hostels. Total cost of the elective is approximately $2500. The majority of students receive partial funding from a travel stipend from the Dean’s Office, and through Global REACH, a University of Michigan Medical School institution that facilitates international initiatives, to help offset the cost of their experience. Global REACH also provides pre-departure planning and logistical support, including information about visas, travel safety, and travel health insurance. In addition, students meet with Dr. Timothy R.B. Johnson who developed the elective experience, and review a powerpoint and guide developed and updated by student participants.

University of Michigan has been successful in it’s commitment to host two Ghanaian medical students for every UMMS student who rotates in Ghana, with approximately ten Ghanaian medical students rotating at University of Michigan annually. Ghanaian medical students participate in 4 week clincial electives as senior medical students. They participate in a shadowing role, and engage in didactic sessions, department seminars, and simulation center experiences. Visiting students rotate in a range of specialities, primarily OBGYN, internal medicine, and surgery. Ghanaian residents and fellows also participate in elective experiences, which range from one to 3 months and are focused on their sub-speciality.

## Methods

This study was granted Not Regulated status by the University of Michigan institutional review board (HUM00170585). Study participants were defined as University of Michigan medical students who completed an international elective in Ghana between March 2006 and June 2017. Following their elective, medical students each complete and submit a reflection report. The report includes background information on the elective, including date and length of the elective, type of elective (research versus clinical), and foreign and UMMS primary mentors. Short-answer questions include a description of the experience, expectations of the student, high points, disappointments, and suggestions for improvement. Reflective questions include the impact of this experience on their interest in future international work and the future practice of medicine. Students were asked whether they would recommend the experience to other students, and had space to write other comments. Logistical information was also collected, including how the elective was identified, funding sources, local housing, and need for language fluency.

A retrospective qualitative analysis of reports was carried out [18]. Thematic analysis was conducted using NVivo 12 (QSR International, Melbourne, Australia). Two reviewers (EL and YS) separately reviewed all narrative short-answer comments. Through incremental and iterative coding of the comments, we arrived at a total of 26 collective keyword-phrases. After our list of generated themes achieved stability, the coding process was repeated for all responses using our final comprehensive list. Our generated codes were used to develop eight key themes. Using Excel (Microsoft Corporation, Redmond, Washington, USA), basic counts and frequencies were calculated from background quantitative data.

## Results

A total of 58 UMMS students completed international electives in Ghana between March 2006 and June 2017. One report was excluded due to no responses, leaving a total of 57 reports available for analysis. The majority of medical students (93%) completed clinical electives, with an average elective lasting 4 weeks (see Table 1). The locations of the clinical electives were primarily at UMMS’s two partner institutions in Kumasi and Accra, Ghana. The majority of clinical electives were done in the Department of OBGYN (72%), with other students rotating with General Surgery, Internal Medicine, and Pediatrics (see Table 1). All students were American citizens, fluent in Engligh.

**Table 1:** Summary of International Elective Experiences

Experience	Frequency
Type of elective	
Clinical	53 (93)
Volunteer	3 (5)
Research	1 (2)
Length of elective, weeks^a^	4 (3-9)
Location of elective^b^	
Accra: Korle Bu Teaching Hospital (University of Ghana)	47 (89)
Kumasi: Komfo Anokye Teaching Hospital (Kwame Nkrumah University of Science and Technology)	5 (9)
Wa: Wa Regional Hospital and Rehabilitation Center	1 (2)
Medical specialty^b^	
Obstetrics and Gynecology	38 (72)
General Surgery	4 (8)
Internal Medicine	8 (15)
Pediatrics	3 (6)

Data presented as n (%) except where noted

^a^Mean (range)

^b^For clinical electives

Importantly, 55 of 57 (96%) of students would recommend their international elective experience to future medical students. When asked if they want to engage in more international work in the future, the vast majority (54/57; 95%) of students responded yes, 2 (3.5%) were unsure, and 1 (2%) responded no.

Benefits of the international elective experience included building cross-cultural relationships, exposure to different healthcare environments, hands-on clinical and surgical experience, and exposure to different medical conditions and patient populations. Impact on their future practice of medicine included motivation for future international work, exposure to limited resources, development of physical exam skills, and professional development (see Table 2). Consistent responses and themes were seen across specialties, clinical sites, and types of elective.

**Table 2:** Thematic Analysis of International Elective Experiences

Theme	Representative quotation
**Benefits of the international elective experience**	
Building cross-cultural relationships	*I enjoyed the time spent with the residents and attending physicians there. They had a lot of very interesting and enlightening insights about medicine globally, the role of countries like the US, and the day to day practice of working as a physician in Ghana.*
Exposure to different healthcare environments	*Although I knew Ghana would be more under-resourced than the US, it was still very eye-opening to see how physicians dealt with these problems first hand. There are also cultural aspects that can only be experienced and learned with immersion into another country. Through this rotation, I feel that I have gained more of an understanding about how to bridge the gap between cultural differences and scientific knowledge.*
Hands-on clinical and surgical experience	*In the operating room, I had the opportunity to first assist in procedures including c-sections and tubal ligations, which allowed me to build basic surgical skills.*
Working with new patient populations/ exposure to different medical conditions	*I was exposed to a wide variety of medical cases that are rare in the United States, including sickle cell disease in pregnancy, malaria, and obstetric fistulas. I also had the opportunity to participate in the care of patients who presented with severe complications and at late stages of diseases that are not commonly encountered in developed countries. Working with patients who presented with malignant reproductive cancers and life-threatening obstetric complications was transformative in my understanding of the importance of cancer screening and prenatal care.*
**Impact on future practice of medicine**	
Motivation for future international work	*“With an interest in Gastroenterology, I became acutely aware of the lack of trained endoscopists in the nation, the limited knowledge in interventional endoscopy, as well as the numbers of patients suffering from chronic hepatitis B now complicated by cirrhosis and end stage liver disease and hepatocellular carcinoma. While the knowledge was disheartening, it provided an opportunity and area which I can now work towards and hopefully one day tangibly impact the lives of these patients. Had it not been for this rotation, I would have still been naive to these issues. I am excited about the potential to be an active participant in the healthcare of this nation and I am now more so than ever, resolved to making sure that I do not become complacent and actually meet these goals.”*
Exposure to limited resources vs overutilization in USA	*“Patients pay for everything out of pocket in Ghana, which means that the physicians there think a lot more about the medications and tests they are ordering, and whether they are really necessary. I hope that, having had this experience, I will remember these two things throughout my residency and not take the technology and resources we have in the US for granted.”*
Professional development	*“This experience has definitely impacted the way I think of myself as a physician. It taught me that being a physician is more than diagnosing and treating diseases. It’s also about being an advocate for your patients and a good steward of resources.”*
Development of physical exam skills	*“Learning how to do a very careful and thorough physical exam was an experience I valued deeply and was a wonderful addition to my American medical school training.”*

Students spontaneously commented on the value that the multi-decade partnership between UMMS and institutions in Ghana added to their experience. Students were able to experience the strength of the Ghana-Michigan longitudinal relationship and better understand the importance and role of these sustained partnerships in global health.

*“The impact of Michigan’s partnership with Ghana was evident on a daily basis, and I came away from this experience with a strong belief in the significance of forming long-term sustainable international partnerships.”*

*“It was incredible to see the depth of Michigan’s collaborations with obstetrics and gynecology training programs in the country.”*

The partnership modeled collaboration and mutual trust, and many students found inspiration for the direction of their own careers. Some students with prior international experiences were able to appreciate the uniqueness of the Ghana-Michigan partnership’s focus on bidirectional exchange.

*“In the past, I’ve been abroad to volunteer and shadow, but my trip to Ghana truly felt like an exchange, since Ghanaian medical students, residents, and faculty come to Ann Arbor to learn about medical practice at the University of Michigan. As a result, the elective reinforced the idea that as a physician, I will be a lifelong learner, as well as collaborator, not only with my patients, but with the colleagues I meet in the United States and abroad.”*

Many students felt that the long-established partnership allowed them to build cross-cultural relationships with peer medical students, residents, and faculty in Ghana. Many of the Ghanaian residents and faculty that supervised visiting Michigan medical students had themselves spent time in Michigan throughout their careers. Students felt they were trusted as a member of the partnership, and were incorporated “...*not as a foreign medical student but as a valuable team member.”*

*“Our presence was welcomed and we were encouraged to share our thoughts about our clinical experiences in Ghana versus in [the] US.”*

*"Easily the best aspect of our experience was the opportunity to sit in on the Ob/Gyn morning meetings - a daily convening of house officers, residents, attendings, midwives and nurses. This meeting provides a forum for discussion of medical issues as well as systemic problems such as a lack of ultrasound gel, medications, or the lack of use of monitoring equipment. We felt privileged to be allowed this intimate view of Ghanaian health care, particularly when, during one particularly difficult meeting, a stranger was asked to step out when he came in part way through. I never expected to see such open discussion of difficult issues, and have not seen anything like it on such a regular basis, involving such a diverse array of providers, at any other time during my medical training.*

Students consistently reported their interactions and relationships with Ghanaian medical students as a high point of their experience.

*"I had the opportunity to work and interact with Ghanaian medical students and faculty and gain and invaluable cross-cultural perspectives on medicine and health care."*

*"... [Students and faculty] went out of their way to make me feel welcomed. I felt like family to them on several occasions."*

*"I loved getting to know the [University of Ghana medical] students on my team; I still keep in touch with them and they made it quite hard to leave."*

Although the experiences were overwhelmingly positive, several common areas for improvement were highlighted. Students suggested that a more streamlined application process and better communication from the Ghanaian host institution prior to departure could have improved the experience. Clearly defining the role of the elective student and establishing expectations for clinical responsibilities were also cited as areas for improvement. Local student exam schedules and physician strikes altered clinical schedules, which was frustrating for some Michigan students. The presence of local medical students was often viewed as a strength, however some Michigan students felt that the number of trainees limited one-on-one time with attendings and opportunities to assist in procedures. Although most healthcare providers were fluent in English, patients often communicated in local languages, and students were challenged to navigate the language barrier. No students experienced significant health issues or adverse events.

## Discussion

Across a decade of engagement in international medical electives in Ghana, University of Michigan medical students reported common benefits of their experiences, including building cross-cultural relationships, exposure to different healthcare environments, hands-on clinical and surgical experience, and working with new patient populations. They also reported important impacts on their future practice of medicine, including motivation for future international work, learning efficient use of limited resources, professional development, and improvement in physical exam skills. To our knowledge, this is the first evaluation of how international medical electives conducted in the context of a long-standing, sustainable academic partnership impact visiting students. These perspectives and skills gained are important in the context of our current healthcare system, with rising costs, poor resource utilization, a growing reliance on technology, and an increasingly global and diverse patient population. Though individual experiences varied, the vast majority of students reported they planned to engage in additional global health work and that they would recommend an international elective to their peers.

Importantly, as these medical student electives exist within the context of decades of bilateral engagement within the Ghana-Michigan partnership [8, 9, 13, 16], students commented specifically on the value that the partnership added to their experience. They felt that the overarching bidirectional exchange allowed them to build cross-cultural relationships and be incorporated as a trusted part of the local clinical team. The partnership modeled collaboration, and many students found inspiration for the direction of their own careers. Global electives within the Ghana-Michigan partnership display many tenets of the WEIGHT (Working Group on Ethics Guidelines for Global Health Training) criteria [19], focusing on ethical global engagement, transparency, reciprocal benefit, and equity.

Global health electives embedded within longstanding partnerships have the potential to better prepare medical students for residency by contributing to AAMC’s Entrustable Professional Activities (EPAs)—particularly “Collaborate as a Member of an Interprofessional Team” and “Identify System Failures and Contribute to a Culture of Safety and Improvement” [20]. Engaging with a new health system can help students to understand how other systems operate and to think more critically about the organization, function, and processes of their own. However, criticism of applying competencies to global health education includes a lack of appreciation of local contexts when developing, applying, and assessing educational competencies to low-resource settings [21]. Sustainable bidirectional relationships, like the Ghana-Michigan partnership, can help address these challenges by involving local partners and approaching the application and evaluation of competencies in a collaborative manner.

In Ghana, UMMS students gained translatable skills that are central to professional development, including patience, versatility, and working with patients and providers from different backgrounds and languages. They learned about the role that physicians can play outside of clinical care, as patient advocates and good stewards of resources. Many were personally challenged to engage as global citizens and to stretch their own comfort zones. Participating in long-term bilateral exchanges that model reciprocity, program management by local physicians, and cultural humility can prepare learners to ethically engage with partners in low-resource settings throughout their careers. This helps ensure that future global health leaders have a collaborative mindset and can cultivate balanced, long-term partnerships.

As with any structured survey, this research is limited by an inability to ask clarifying and follow-up questions. However, the selected questions were open-ended and encouraged reflection. The survey was completed retrospectively, but within a short time frame after completion of the elective, thus limiting recall bias. Participants were limited to a single university, which was done to take advantage of learning from a unique established international partnership. Information on participants’ age, gender, and past international experience was not collected, which could impact responses. Responses were collected from all students who completed an international elective in Ghana over a decade, minimizing selection bias and supporting diversity of experiences.

Further research is needed on longterm followup of medical student participants in international electives to explore impact on their career trajectories and engagement in future global work. In addition, the perspective of Low and Middle Income hosts who facilitate electives for American medical students has not been assessed.

## Conclusions

In conclusion, embedding clinical rotations within a well-established relationship such as the one shared between the University of Michigan and Ghanaian medical schools provides valuable experiences for trainees and also contributes to the ultimate sustainability of the partnership. Lessons learned from this research can be used to guide the development and improvement of medical student international experiences to develop globally minded future physicians who engage in ethically and productive ways. The University of Michigan’s collaboration with institutions in Ghana demonstrates a reciprocal partnership that supports bidirectional medical and cultural exchange and promotes equity in global medical education.

## Data Availability

The datasets generated and/or analyzed during the current study are not publicly available due to the survey being conducted internally within our institution, but are available from the corresponding author on reasonable request.

## Abbreviations

AAMC: Association of American medical colleges
OBGYN: Obstetrician gynecologist
UMMS: University of Michigan medical school
EPA: Entrustable professional activity
